# Medical Society of the County of Erie

**Published:** 1894-02

**Authors:** Eli H. Long

**Affiliations:** Secretary


### Society Proceedings.

### MEDICAL SOCIETY OF THE COUNTY OF ERIE.

*Annual Meeting, January 9, 1894.*

Reported by ELI H. LONG, M. D., Secretary.

THE annual meeting of the Medical Society of the County of Erie was called to order by the President, Dr. John Parmenter, at 10.40 A. M.

The minutes of the last semi-annual and two special meetings were read and approved.

The Membership Committee reported favorably upon the following-named applicants for membership, and, upon motion, they were unanimously elected: Drs. Albert T. Lytle, Ada C. Latham, Harry Mead, Dewitt H. Sherman, Horace Clark, Charles S. Jewett, A. W. Bayliss, Cyrus S. Siegfried, and Francis T.

Metcalfe. The committee further reported that Francis A. Drake and Charles H. Perry had failed to exhibit their diplomas, therefore could not be reported upon at this meeting.

Applications for membership by the following-named physicians were presented and, according to rule, were laid over until the next regular meeting: William Meisburger, Maud J. Frye, Helene J. C. Kuhlmann, Ludwig Schroeter, William G. Taylor and William C. Fritz.

Dr. Wm. Warren Potter moved to postpone the President's address until after completion of the executive business. Adopted.

The Secretary moved that communications be now read. Adopted.

Communications from the National Quarantine Committee and of the New York Academy of Medicine, relating to the bill proposing to establish a Bureau of Public Health, were read, and, in addition, the Secretary reported that, upon suggestion by the same committee, the President and Secretary had transmitted letters to the members of Congress from this State and likewise to the Committee on Epidemic Diseases of the United States Senate.

Dr. Hopkins moved that the action of the officers as reported be endorsed. Adopted.

A further communication from the New York Academy of Medicine, relating to the coroner question, was received and filed.

The resignations of Drs. S. W. and Mary Wetmore from membership, on account of removal from the county, were read and, upon motion, the same were accepted.

A communication from Dr. J. F. Krug, in reference to illegal practitioners, was read.

Dr. Walsh moved that the communication be received with thanks to Dr. Krug.

The Secretary moved to amend by adding that the communication be referred to the new Board of Censors. The motion as amended was adopted.

At the suggestion of the President, miscellaneous business was taken up at this time.

Dr. E. H. Long, chairman of the committee on revision of the by-laws, then made the final report of the committee, which was in form of printed proofs of the by-laws as recommended by the committee. The report was considered section by section, and a few amendments thereto adopted. The chief amendment was offered by Dr. W. W. Potter, and consisted of a section providing for the appointment of a standing committee on hygiene. After amendment, the report was adopted as a whole and declared to be the by-laws of the society.

It was then moved that the Secretary be authorized to have the by-laws printed and that a copy be sent to each member. Adopted.

The Secretary asked whether, in the revised list of members, the names of non-residents who were formerly members of the society should appear. It was decided that only names of residents of the county could properly appear, and the Secretary was instructed accordingly.

The Secretary also asked what should be done with the resignation of Dr. Geo. H. McMichael from membership, which had been presented at a previous meeting. Dr. Callanan moved that the resignation be accepted. Carried.

Dr. J. S. Porter moved that a committee of three be appointed to draft a bill to be presented to the Legislature, which shall require every practising physician to qualify before the State Board of Medical Examiners. Dr. B. G. Long moved to amend by making it the duty of the committee to petition the Regents to use all possible means at their command to regulate the practice of medicine. The amendment was seconded by Dr. L. Howe, and adopted. The motion as amended was then carried.

The Chair appointed as such committee: Drs. James S. Porter, H. R. Hopkins and W. W. Potter.

The election of officers was then taken up.

Dr. Walsh moved that a committee of five be appointed to nominate officers for the ensuing year. Carried.

The Chair appointed as such committee: Drs. H. E. Hayd, Henry Lapp, B. G. Long, R. L. Banta and E. L. Frost.

After a conference the committee reported the following list of nominees: President, Dr. Wm. H. Gail; Vice-President, Dr. F. W. Bartlett; Secretary, Dr. Franklin C. Gram; Assistant Secretary, Dr. George F. Cott; Treasurer, Dr. Edward Clark; Assistant Treasurer, Dr. Eugene Smith; Librarian, Dr. Wm. C. Callanan; Committee on Membership, Drs. Jno. A. Pettit, Jas. W. Putnam and G. W. MacPherson; Censors, Drs. J. F. Krug, Jas. S. Porter, J. H. Potter, Henry Lapp, A. L. Benedict; Delegate to Medical Society of the State of New York, to fill vacancy, Dr. John H. Pryor.

The Secretary was directed to cast the ballot of the society for the nominees, and they were then declared elected.

The President then appointed the committee on Hygiene to consist of Drs. H. R. Hopkins, Edward Clark, E. C. W. O'Brien, W. W. Potter and W. D. Greene.

The President reported the library now available in one of the alcoves of the library of the Uuiversity of Buffalo.

The Librarian's report for the year is as follows :

*To the Medical Society of the County of Erie :*

Up to the time of the removal of your library from the old college, there were no applications to me for books. In accordance with the desire of the society that the books should be available ; and with the authority from the resolution passed at the last annual meeting, constituting the President and Librarian a committee with power of selection and location, the committee moved the books to the new college. An alcove, nicely cased and shelved, was given for books, and therein they have been placed. The services of the librarian of the college library were given me, and she has arranged the books.

Owing to almost continuous absence from the city, and now permanent removal, I have been unable to catalogue the books, nor am I able to report the degree of their use.

I wish to acknowledge the courtesy of the college authorities, and especially of the secretary and the librarian, and I thank them for the same in this report to you.

I would suggest that the society empower its librarian to make such arrangements with the college librarian as shall render the books most available, as often members cannot afford time to hunt up the librarian or go to his office.

I thank you for the honor conferred upon me in the election as your librarian in the last two years.

Respectfully submitted,

CHARLES A. RING,

*Librarian.*

The Treasurer's annual report, showing a balance on hand of $148.92, was then presented and referred to an auditing committee, consisting of Drs. J. W. Putnam and B. G. Long.

The Auditing Committee reported the Treasurer's accounts correct, and they recommended that the Treasurer be instructed to notify all members who are three years in arrears for dues, that unless their dues are paid, their names will be dropped from the membership list of the society.

The report of the Auditing Committee, including their recommendation, was unanimously adopted.

The President's valedictory was then presented and read.

It was moved that the address be published in The Buffalo Medical and Surgical Journal. Carried.

Dr. W. W. Potter moved that the Secretary and Treasurer be each paid the sum of $50.00 for services during the past year. Carried.

Dr. Callanan moved that the Board of Censors be authorized to employ an attorney when necessary, but that the expense shall not exceed $100.00 for one year. Carried.

The society adjourned at 1.10 p. m.

